# A comprehensive framework for analysis of microRNA sequencing data in metastatic colorectal cancer

**DOI:** 10.1093/narcan/zcab051

**Published:** 2022-01-14

**Authors:** Eirik Høye, Bastian Fromm, Paul H M Böttger, Diana Domanska, Annette Torgunrud, Christin Lund-Andersen, Torveig Weum Abrahamsen, Åsmund Avdem Fretland, Vegar J Dagenborg, Susanne Lorenz, Bjørn Edwin, Eivind Hovig, Kjersti Flatmark

## Abstract

Although microRNAs (miRNAs) contribute to all hallmarks of cancer, miRNA dysregulation in metastasis remains poorly understood. The aim of this work was to reliably identify miRNAs associated with metastatic progression of colorectal cancer (CRC) using novel and previously published next-generation sequencing (NGS) datasets generated from 268 samples of primary (pCRC) and metastatic CRC (mCRC; liver, lung and peritoneal metastases) and tumor adjacent tissues. Differential expression analysis was performed using a meticulous bioinformatics pipeline, including only bona fide miRNAs, and utilizing miRNA-tailored quality control and processing. Five miRNAs were identified as up-regulated at multiple metastatic sites Mir-210_3p, Mir-191_5p, Mir-8-P1b_3p [mir-141–3p], Mir-1307_5p and Mir-155_5p. Several have previously been implicated in metastasis through involvement in epithelial-to-mesenchymal transition and hypoxia, while other identified miRNAs represent novel findings. The use of a publicly available pipeline facilitates reproducibility and allows new datasets to be added as they become available. The set of miRNAs identified here provides a reliable starting-point for further research into the role of miRNAs in metastatic progression.

## INTRODUCTION

Colorectal cancer (CRC) is a heterogeneous disease and a leading cause of cancer-related deaths worldwide (1), and metastatic progression to the liver, lungs and peritoneal surface remains the primary cause of CRC-related mortality. Metastasis is a complex process where cancer cells undergo adaptation to enable survival and establishment of tumors in organs with very different microenvironments (2,3). The genomic and transcriptomic changes in metastatic CRC (mCRC) remain incompletely understood, particularly in the context of organ-specific metastasis (4).

MicroRNAs (miRNAs) are evolutionary ancient post-transcriptional gene regulators that are involved in numerous biological processes and are molecular players in human disease, including cancer (5,6). MiRNAs can be extracted from tissues and body fluids, and because of their remarkable chemical stability and the availability of sensitive detection methods, miRNAs have been suggested as cancer biomarkers (7–10). However, to date, no miRNAs have been clinically implemented as biomarkers of CRC (11,12). In mCRC, in particular, there is little consensus regarding which miRNAs are up- and down-regulated, limiting our understanding of their role in metastatic progression(11).

The lack of consensus likely reflects some well-known caveats related to analysis of miRNAs in human disease. Non-miRNA sequences have been incorrectly annotated as miRNA genes (13), and a bioinformatics workflow specifically tailored for the analysis of miRNAs in bulk tissue samples has not been available. Furthermore, differential expression analysis has been performed without ensuring the presence of physiologically relevant tissue expression levels (14). Also, accounting for differences in cellular composition is important, since many miRNAs are exclusively expressed in particular cell types or at specific developmental time points (15–18), likely confounding analysis of bulk tissue samples. Finally, to elucidate the role of miRNAs in mCRC, failure to consider differences related to metastatic location, and differences in normal background expression, may have contributed to inconsistent results (19,20).

To overcome these challenges, the publicly available, manually curated miRNA gene database MirGeneDB (mirgenedb.org), was used as miRNA reference (21), and a novel bioinformatics pipeline was developed. The bioinformatics work-flow included use of the miRTrace software as a universal quality control pipeline specifically for miRNA next-generation sequencing (NGS) data (22) with subsequent processing using miRge3.0 (23). A strict cut-off of 100 reads per million (RPM) was applied as the minimum expression level for physiological relevance (14). Taking cell-type specific miRNA expression into account, metastatic samples from different sites were independently analyzed, which allowed correction for different background expression levels at the individual sites. Existing publicly available miRNA-sequencing (miRNA-seq) patient derived datasets, combined with novel miRNA-seq datasets from pCRC and mCRC with normal adjacent tissues, were analyzed, after quality control totaling 268 datasets. Using this unbiased analytical approach, multiple miRNAs were found to be differentially expressed in mCRC. This finding partially overlaps with previous reports, but also includes several miRNAs not previously identified in this context.

## MATERIALS AND METHODS

### Patient samples

Tissue samples were obtained from study specific biobanks: pCRC and tumor adjacent colorectum (nCR) samples were from the LARC-EX study (NCT02113384); liver metastases (mLi) and tumor adjacent liver (nLi) samples from the OSLO-COMET trial (NCT01516710); lung metastases (mLu) and tumor adjacent lung (nLu) samples from our lung metastasis biobank (S-06402b) and peritoneal metastases (PM) samples from the Peritoneal Surface Malignancies biobank (NCT02073500). The studies were approved by the Regional Ethics Committee of South-East Norway, and patients were included following written informed consent. Patient samples were collected at the time of surgery and were snap frozen in liquid nitrogen at the time of collection and stored at −80°C. Samples were prepared and processed as described in (24).

### RNA extraction and NGS

RNA was extracted using Qiagen Allprep DNA/RNA/miRNA universal kit, which simultaneously isolates genomic DNA and total RNA. RNA concentration was evaluated using a NanoDrop spectrophotometer (ThermoFisher, Waltham, Massachusetts, USA) and RNA integrity was evaluated using the Bioanalyzer RNA 6000 Nano kit (Agilent Technologies, Santa Clara, California, USA). MiRNA NGS libraries were then prepared using TruSeq Small RNA Library protocol and sequences using HiSeq 2500 High Throughput Sequencer (all from Illumina, San Diego, California, USA).

### Identification of published NGS datasets

A literature search was conducted for the terms ‘microRNA + CRC + next generation sequencing’ in different variations, and reviews were studied (25,26). Publicly available datasets were downloaded from European Genome-phenome Archive (EGA), the Sequence Read Archive (SRA) and the Gene Expression Omnibus (GEO) (19,27–30).

### Data processing, read alignment and gene counting

All datasets were processed using the same pipeline. miRTrace (22) was used for preprocessing and quality control (QC) of raw data (FASTQ files). Briefly, low-quality reads, defined as reads where <50% of nucleotides had a Phred quality score >20 were discarded. 3p adapter sequences were trimmed, and reads made up of repetitive elements and reads <18 nt were removed. After miRTrace QC, samples were excluded if <25% of reads were between 20 and 25 nt, if >75% of reads were discarded, or if <10% of reads were identified as miRNA. If >50% of the datasets in a study failed the QC criteria, or if significant contamination was detected, it was excluded. After QC, raw data processing, read alignment and gene counting was performed using miRge3.0 (23), with MirGeneDB2.0 (21) as reference. To account for cross mapping of miRNA genes, miRge3.0 merges miRNA genes with very similar sequences into one annotation, reducing 537 human miRNA annotations in MirGeneDB2.0 to a total of 389 unique annotations.

### Analysis of global miRNA expression

For data visualization of global miRNA expression, read counts were normalized using the Variance Stabilizing Transformation() (VST) function from the DESeq2 package (31). The dimensionality reducing algorithm uniform manifold approximation and projection for dimension reduction (UMAP) was used to visualize the similarity of datasets on the global miRNA expression level. The UMAP algorithm reduces the 389 dimensions of unique miRNA genes into two dimensions for visualization (32). The UMAP R package was used, and datasets were annotated by tissue.

### Differential expression analysis

The differential expression analysis can be viewed in the supplementary R-markdown file (Supplementary File S3).

Differential expression analysis was performed using DESeq2 (version 1.26) (31), which estimates Log2 fold change (LFC) and its standard error (SE), using raw, non-normalized read counts as input. Hypothesis testing was performed by a Wald test against the null hypothesis LFC = 0, followed by the Benjamini-Hochberg procedure to correct for multiple hypothesis testing, applying a false discovery rate (FDR) threshold of <0.05. The LFC shrinkage function in DESeq2, lfcShrink(), was enabled, to shrink fold changes for miRNAs with higher variance. Differentially expressed miRNAs were filtered for relevance by requiring an LFC >0.58 or LFC < -0.58, and also requiring that at least one of the compared tissues had a mean expression >100 RPM, a level previously suggested as minimal cut-off for physiological activity (14). MiRNAs known to be cell-type specific (16) were also labeled for each miRNA. Information regarding normal tissue background expression levels was obtained by analyzing differential expression between nCR versus nLi and nLu datasets, and pCRC versus nLi and nLu datasets. Then, in the mCRC versus pCRC differential expression analysis, miRNAs differentially expressed in the same direction in the corresponding normal tissue were not considered differentially expressed in the respective metastatic site. For the PM tissue datasets, where no tumor adjacent tissue was available, the union of nLi and nLu background expression was used.

### qPCR validation

Eleven additional randomly chosen mLi and 11 pCRC tissue samples were selected for qPCR validation of increased expression of Mir-210_3p in mLi compared to pCRC. Synthetic RNA Spike-Ins UniSp2, UniSp4 and UniSp5 (Qiagen, Düsseldorf, Germany Cat. No. 339390) were added pre-isolation. RT-PCR was done with miRCURY LNA RT Kit (Qiagen Cat. No. 339340), adding UniSp6 and cel-miR-39–3p RNA Spike-Ins (Qiagen Cat. No 339390). qPCR was done using Qiagen miRCURY SYBR Green Kit (Qiagen Cat. No. 339345) and miRCURY LNA miRNA PCR Assays (Qiagen Cat. No. 339306). Mir-103 (Qiagen primer Cat. No. YP00204306) was used as reference miRNA, while the Mir-210_3p primer was Qiagen Cat. No. YP00204333. Two PCR replicates were run per primer assay. Mir-210_3p Cq values were normalized to the reference miRNA to obtain the dCq value. Welch two-sided *t*-test was used to compare the dCq values, to test if the expression levels were different between mLi and pCRC. (Supplementary File S5).

### Analysis of cell-type specific miRNAs

Two analyses were performed to assess differences in expression levels of 45 previously validated cell-type specific miRNAs (16), and thereby infer differences in cell composition in the tissues. A heatmap using z-scores of RPM values per miRNA was made to illustrate the relative expression of each miRNA in the tissues (Figure 4A). Principle component analysis (PCA) plots were made with the FactoMineR PCA() function to illustrate which miRNAs were more or less prevalent in each tissue. Welch two-sided *t*-test comparing mean VST values between two groups of tissues was done to assess if the observed differences in cell-type specific miRNA levels were statistically significant (Supplementary File S4).

### Gene set enrichment analysis

Gene set enrichment analysis (GSEA) was performed using RBiomirGS (33) on GO Molecular Function, Cellular Component and Biological Process and KEGG pathways, downloaded from https://www.gsea-msigdb.org/gsea/msigdb/collections.jsp. miRNA to mRNA predicted interactions were combined with data from the miRNA differential expression analysis (DESeq2 estimated LFC and FDR values for each mCRC site versus pCRC). The input values to RBiomirGS were corrected for normal background expression, reducing LFC towards 0 and increasing FDR toward 1, depending on the magnitude of the normal background. All miRNAs were then given an S_miRNA_ score -log10 *P*-value * sign(log_2_FC)., and for each mRNA, a S_mRNA_ score was calculated by summing up the S_miRNA_ scores of all the predicted miRNA to mRNA interactions. The S_mRNA_ scores were then used to perform logistic regression, which provides likelihoods of gene sets being more or less suppressed due to differential expression of miRNAs.

## RESULTS

### NGS data collection and processing

New NGS datasets were successfully generated from 85 samples: pCRC (*n* = 3), nCR (*n* = 3), mLi (*n* = 19), nLi (*n* = 9), mLu (*n* = 25), nLu (*n* = 7) and PM (*n* = 20). From five studies, previously published datasets containing NGS analyses of miRNA in pCRC and mCRC were also included (19,27–30) while data from four published studies were not accessible (34–37). In total, 350 NGS datasets were subjected to quality assessment using the miRTrace pipeline. The majority of the new NGS datasets and datasets from three previously published studies (19,29,30) fulfilled the quality control criteria. Two studies were excluded, one because of low quality reads in the majority of samples (38), the other because of contamination of reads from other organisms (28) (Supplementary File S1). After quality control, a total of 268 NGS datasets remained for further analysis, including pCRC (*n* = 120), nCR (*n* = 25), mLi (*n* = 35), nLi (*n* = 20), mLu (*n* = 28), nLu (*n* = 10) and PM (*n* = 30). These datasets were then successfully processed and mapped to MirGeneDB (21) using miRge3.0 (23). Summary of clinicopathological parameters for the included datasets, where these were available, along with summary of tumor content for each tissue and study, are shown in Supplementary Table S1.

### Global miRNA expression

**Figure 1.**
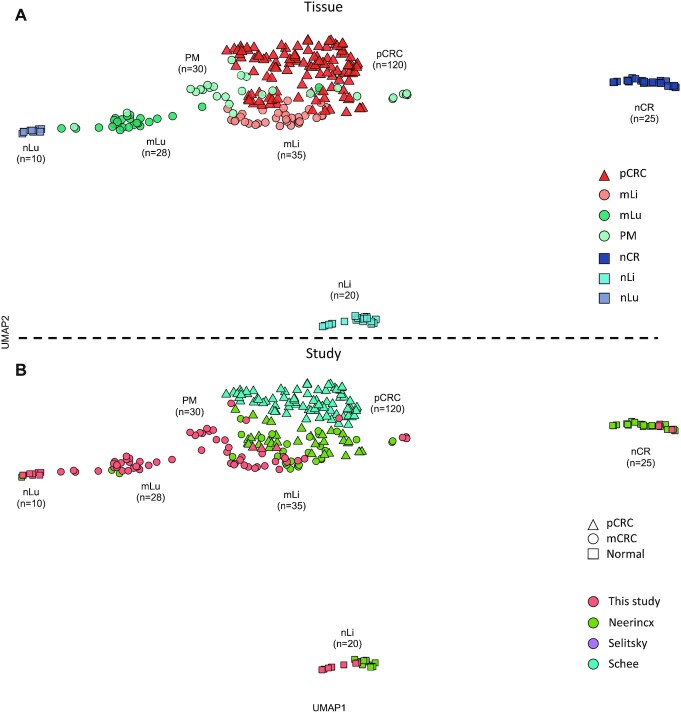
Global miRNA expression according to tissue of origin. (**A**) UMAP cluster plot based on global miRNA expression, normalized by VST, from the DESeq2 bioconductor package, annotated by tissue of origin. (**B**) UMAP cluster plot based on global miRNA expression, normalized by VST, from the DESeq2 bioconductor package, annotated by study of origin. Uniform approximation and projection for dimension reduction (UMAP); varianceStabilizingTransformation (VST); primary colorectal cancer (pCRC); normal colorectal tissue (nCR); CRC liver metastasis (mLi); normal adjacent liver tissue (nLi); CRC lung metastasis (mLu); normal adjacent lung tissue (nLu); CRC peritoneal metastasis (PM).

When analyzing global miRNA expression using UMAP, the datasets clustered according to the tissue of origin (Figure 1A). The normal tissues (nLi, nLu and nCR) formed distinct clusters reflecting the unique transcriptional profiles of these organs. nLi and nCR clustered separately from the corresponding tumor tissues, while nLu clustered close to the mLu tissue datasets. Furthermore, study of origin for the normal tissues had no effect on their clustering (Figure 1B). In general, the pCRC and mCRC dataset clusters were less homogeneous than the normal tissue counterparts, but with exception of the PM datasets, which exhibited considerable overlap with the other malignant datasets, the pCRC, mLi and mLu datasets all formed distinct clusters. Study of origin also did not appear to impact clustering of mCRC (Figure 1B).

### Differential miRNA expression between pCRC and nCR

Thirty-two miRNAs were up-regulated and 35 miRNAs were down-regulated when comparing pCRC to nCR. Among the differentially expressed miRNAs were many well-known oncomiRs, including Mir-21_5p, multiple MIR-17 family members, Mir-31, Mir-221 (up-regulated) and Mir-8-P1b_3p (miR-141) (down-regulated). Several cell-type specific miRNAs were also detected at different levels in the two tissues, including higher levels of Mir-17-P1a/P1b_5p (mir-17; CD14+ monocytes) and Mir-223_3p (dendritic cells), and lower levels of Mir-486_5p and Mir-451_5P (red blood cells), the Mir-143_5p and Mir-145_5p (mesenchymal cells), Mir-150_5p (lymphocytes), Mir-375_3p and Mir-192-P1_5p (epithelial cells) and Mir-342_3p (dendritic cells, lymphocytes and macrophages) (16). For a complete overview of differentially expressed miRNAs between pCRC and nCR, see Supplementary File S3.

### Differential expression analysis identifies miRNA expression alterations in mCRC

**Figure 2.**
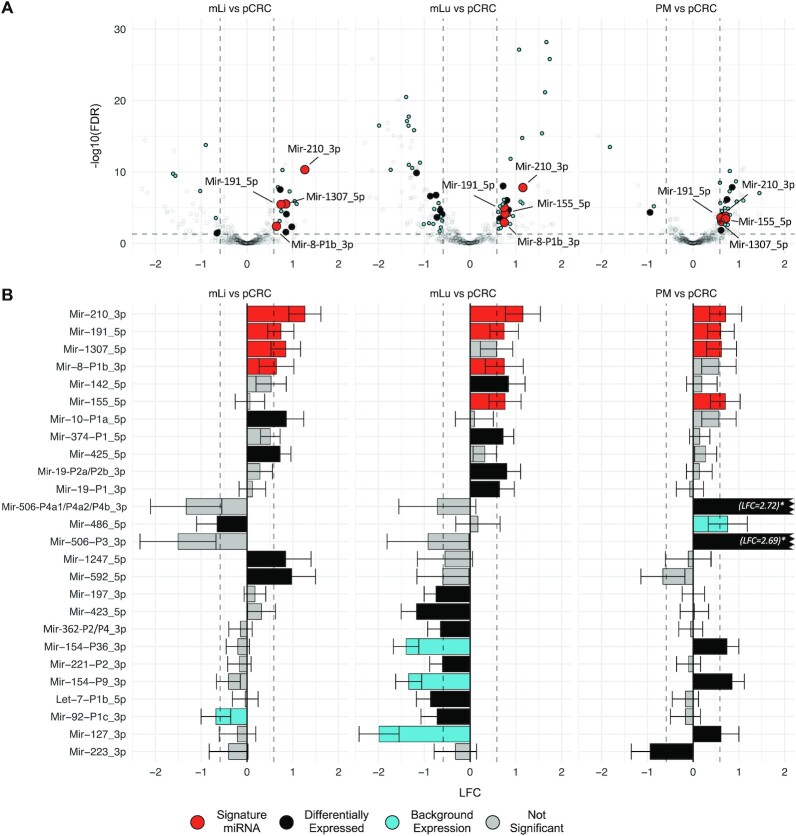
Differential expression analysis identifies miRNAs associated with metastatic colorectal cancer. (**A**) Volcano plots showing differentially expressed miRNAs in mLi, mLu and PM compared to pCRC. The horizontal axis shows LFC relative to pCRC, while the vertical axis shows -log10 FDR. The identified miRNAs were differentially expressed between pCRC and mCRC, with expression levels >100 RPM in one of the tissues, and results were corrected for tumor adjacent background expression. (**B**) Bar plots of LFC of differentially expressed miRNAs in pCRC compared to the individual metastatic sites. Log2 fold change (LFC); false discovery rate (FDR); reads per million (RPM); primary colorectal cancer (pCRC); normal colorectal tissue (nCR); CRC liver metastasis (mLi); normal adjacent liver tissue (nLi); CRC lung metastasis (mLu); normal adjacent lung tissue (nLu); CRC peritoneal metastasis (PM).

**Figure 3.**
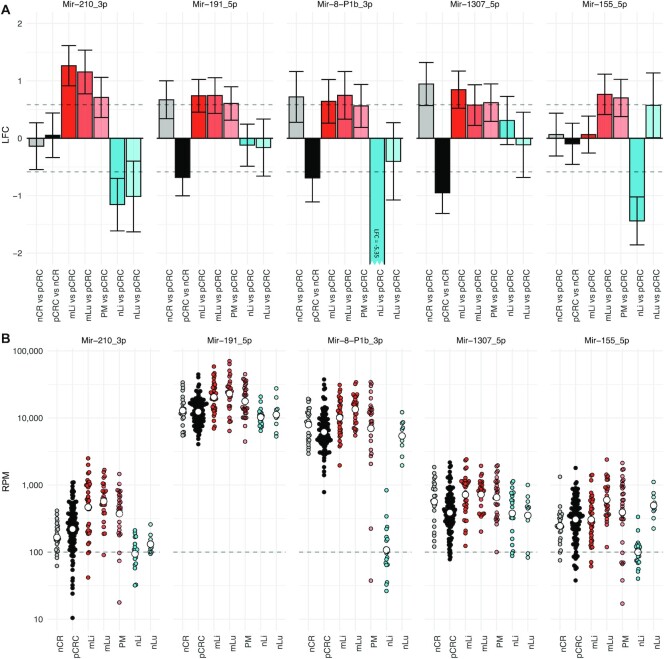
Differential expression analysis identifies five miRNAs up-regulated at multiple metastatic sites. (**A**) Bar plots showing LFC of the five miRNAs that were differentially expressed between pCRC and at least two mCRC sites. Mir-210_3p and Mir-191_5p were up-regulated at all three metastatic sites, while, Mir-8-P1b_3p was up-regulated in mLi and mLu, Mir-1307_5p in mLi and PM, and Mir-155 in mLu and PM, after correcting for multiple hypothesis testing. Error bars represent 95% confidence intervals from DESeq2 estimated SE of the LFC. (**B**) Scatter plots of Log10 RPM in each tissue. Log2 fold change (LFC); reads per million (RPM); standard error (SE) primary colorectal cancer (pCRC); normal colorectal tissue (nCR); CRC liver metastasis (mLi); normal adjacent liver tissue (nLi); CRC lung metastasis (mLu); normal adjacent lung tissue (nLu); CRC peritoneal metastasis (PM).

After performing site specific differential expression analysis and correcting for background expression, a total of 26 miRNAs were identified as differentially expressed in one or more of the metastatic tissues compared to pCRC (Figure 2 and Table 1). Two miRNAs, Mir-210_3p and Mir-191_5p, were up-regulated at all three metastatic sites. In addition, three miRNAs were up-regulated at two of the three sites; Mir-8-P1b_3p in mLi and mLu, Mir-1307_5p in mLi and PM, and Mir-155 in mLu and PM (Figure 3). All these miRNAs were expressed well above the threshold for biological significance, with Mir-191_5p and Mir-8-P1b_3p being expressed at particularly high levels (>1000 RPM).

**Table 1.** Differentially expressed miRNAs in mCRC compared to pCRC according to metastatic site. Reads per million (RPM); log2 fold change (LFC) estimated using DESeq2; standard error (SE); primary colorectal cancer (pCRC); normal colorectal tissue (nCR); CRC liver metastasis (mLi); normal adjacent liver tissue (nLi); CRC lung metastasis (mLu); normal adjacent lung tissue (nLu); CRC peritoneal metastasis (PM); false discovery rate (FDR)

		mLi vs pCRC			mLu vs pCRC			PM vs pCRC		
MirGeneDB ID (miRBase ID)	pCRC RPM	RPM	LFC (SE)	FDR	RPM	LFC (SE)	FDR	RPM	LFC (SE)	FDR
Mir-191_5p	13630	23253	0.74 (0.15)	3.67E-06	26989	0.74 (0.16)	9.81E-06	20429	0.61 (0.15)	2.42E-04
Mir-210_3p	291	685	1.26 (0.18)	4.73E-11	693	1.15 (0.19)	1.55E-08	491	0.71 (0.18)	3.37E-04
Mir-1307_5p	492	887	0.85 (0.17)	2.99E-06				797	0.62 (0.17)	8.29E-04
Mir-8-P1b_3p (mir-141–3p)	7518	11957	0.64 (0.19)	4.07E-03	15111	0.75 (0.21)	1.12E-03			
Mir-155_5p	387				733	0.76 (0.18)	7.24E-05	654	0.70 (0.17)	1.59E-04
Mir-10-P1a_5p (mir-10a-5p)	97123	164925	0.86 (0.19)	8.04E-05						
Mir-592_5p	75	137	0.98 (0.27)	4.88E-03						
Mir-1247_5p	69	127	0.85 (0.28)	2.58E-02						
Mir-425_5p	700	1117	0.73 (0.12)	2.90E-08						
Mir-486_5p	2013	1489	-0.66 (0.23)	3.47E-02						
Mir-142_5p	3453				7117	0.84 (0.18)	2.01E-05			
Mir-19-P2a/P2b_3p	1297				2345	0.80 (0.15)	9.38E-07			
Mir-374-P1_5p	115				217	0.72 (0.12)	9.07E-09			
Mir-19-P1_3p	379				597	0.65 (0.16)	3.42E-04			
Mir-423_5p	433				202	-1.17 (0.17)	1.35E-10			
Let-7-P1b_5p	1057				650	-0.86 (0.16)	2.55E-07			
Mir-197_3p	181				113	-0.74 (0.13)	1.76E-07			
Mir-92-P1c_3p	1398				981	-0.72 (0.18)	2.22E-04			
Mir-362-P2/P4_3p	441				302	-0.65 (0.14)	2.36E-05			
Mir-221-P2_3p	1633				1101	-0.60 (0.14)	8.50E-05			
Mir-506-P3_3p	6							112	2.69 (0.29)	6.35E-09
Mir-506 P4a1/P4a2/P4b_3p	14							191	2.72 (0.29)	5.75E-11
Mir-154-P9_3p	253							455	0.85 (0.14)	1.43E-08
Mir-127_3p	2263							2888	0.61 (0.20)	1.42E-02
Mir-154-P36_3p	195							330	0.74 (0.13)	7.13E-07
Mir-223_3p	619							280	-0.94 (0.21)	4.62E-05

In addition, several miRNAs were up- or down-regulated at individual metastatic sites. In mLi, four miRNAs were identified as up-regulated, Mir-10-P1a_5p, Mir-592_5p, Mir-1247_5p and Mir-425_5p, while Mir-486_5p was down-regulated. Of these, Mir-10-P1a_5p was expressed at exceptionally high levels, with 97 123 RPM in pCRC and 164 925 RPM in mLi. In mLu Mir-19-P1_3p, Mir-19-P2a/P2b_3p, Mir-374-P1_5p and Mir-142_5p were up-regulated while Mir-423_5p, Let-7-P1b_5p, Mir-197_3p, Mir-92-P1c_3p, Mir-362-P2/P4_3p and Mir-221_3p were down-regulated. In PM Mir-506-P3_3p, Mir-506-P4a1/P4a2/P4b_3p, Mir-154-P9_3p and Mir-127_3p, Mir-154-P36_3p were up-regulated and Mir-223_3p were down-regulated.

### qPCR validation

qPCR analysis of randomly selected mLi (*n* = 11) and pCRC (*n* = 11) samples not included in the previous NGS analysis replicated the findings from the NGS data (Figure 5), validating up-regulation of Mir-210_3p in mLi compared to pCRC. Welch two-sided *t*-test on dCq values, *t*-statistic = -2.25, degrees of freedom = 19.95, *P*-value = 0.036.

### Analysis of cell-type specific miRNAs

**Figure 4.**
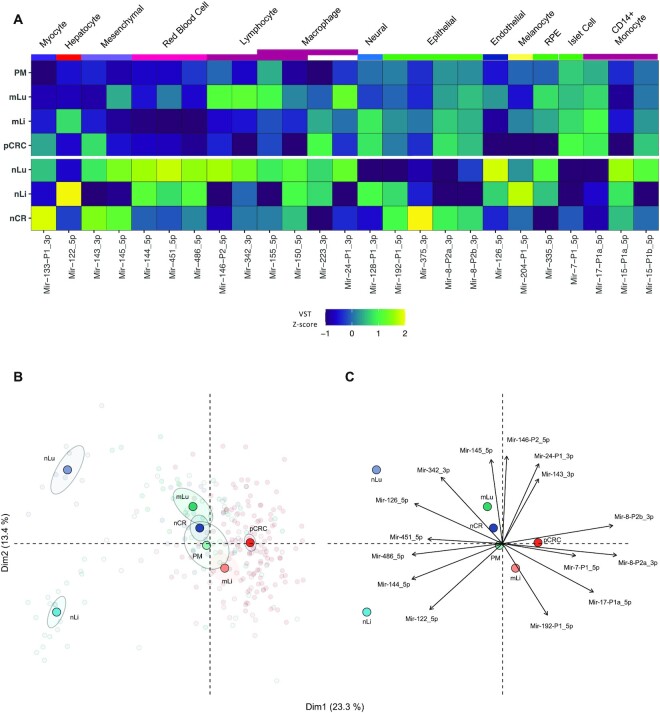
Cell-type specific miRNAs provide insight into the cellular composition of tissues. (**A**) Heatmap illustrating the expression of 25 cell-specific miRNAs for each tissue that had mean expression >100 RPM in at least one tissue. The color scale indicates the *z*-score of RPM for each miRNA. (**B**) PCA plot based on analysis of 25 cell-specific miRNA with expression >100 reads per million, VST normalized, colored by tissue. Transparent points are individual samples, solid points represent the statistical mean and shaded ellipses is 95% confidence of the mean of each tissue. (**C**) The correlation circle shows PCA loadings, which are the direction and relative contribution the top 15 cell-type specific miRNAs had on clustering of each tissue. Reads per million (RPM); principal component analysis (PCA); varianceStabilizingTransformation (VST); primary colorectal cancer (pCRC); normal colorectal tissue (nCR); CRC liver metastasis (mLi); normal adjacent liver tissue (nLi); CRC lung metastasis (mLu); normal adjacent lung tissue (nLu); CRC peritoneal metastasis (PM).

Of the 45 previously validated cell-type specific miRNAs, only 25 had a mean expression >100 RPM in at least one of the tissues and formed the basis for the following analysis. The relative expression levels of cell-type specific miRNAs showed clear differences between the tissues, particularly prominent for the tumor adjacent tissues (Figure 4A). For instance, hepatocyte specific Mir-122_5p was detected at high and moderate levels in nLi and mLi, respectively, and at extremely low levels in the other tissues. Also, Mir-143_3p and Mir-145_5p was detected at higher levels in nCR and nLu relative to the other datasets (*P* = 4.38E-02 and *P* = 3.42E-04, respectively). The epithelial cell specific Mir-8-P2a_3p and Mir-8-P2b_3p were detected at higher levels in the intestinal epithelial-derived tissues compared to nLi and nLu (*P* = 2.20E-16 and *P* = 2.24E-16, respectively). In line with these findings, principal component analysis (PCA) (Figure 4B) showed distinct clusters for nLi and nLu, whereas nCR clustered closer to pCRC and the metastatic tissues. The correlation circle (Figure 4C) shows the loading of the PCA, indicating the direction and relative contribution of the 15 cell-type specific miRNAs that contributed most to the clustering of each tissue.

**Figure 5.**
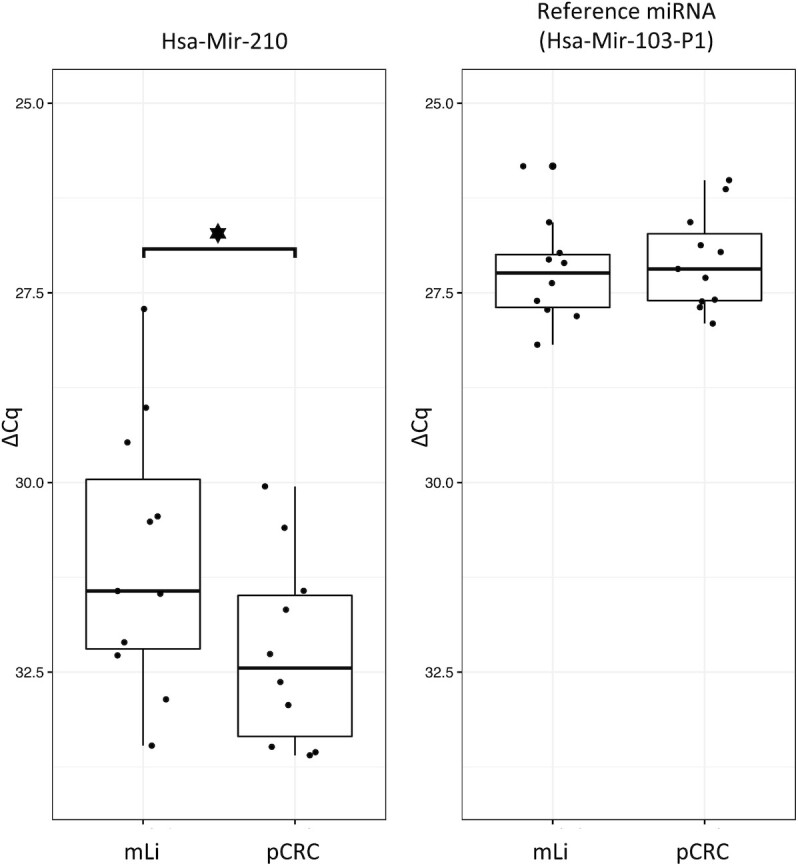
qPCR of Mir-210 in mLi versus pCRC. Mir-103-P1 was used as reference miRNA. Δ*C*q values are plotted on the *y*-axis; these are the *C*q values from Mir-210 normalized to the reference gene Mir-103-P1. CRC liver metastasis (mLi); primary colorectal cancer (pCRC).

### Gene set enrichment (GSE) analysis

RBiomirGS analysis revealed multiple GO terms and KEGG pathways that were predicted to be either more or less repressed because of differential expression of miRNAs between pCRC and mCRC. Among terms predicted to be less repressed, several were common to mLi and mLu. These included the five Molecular Function GO terms: Transcription Coregulator Activity, Transcription Coactivator Activity, Transcription Regulator Activity, mRNA Binding and Poly Purine Tract Binding. Also estimated to be less repressed were the eight Cellular Component GO terms, including Transcription Repressor Complex, Caveola, Transferase Complex, Golgi Apparatus, Glutamatergic Synapse, Catalytic Complex, Nuclear Envelope and Transcription Regulator Complex. Furthermore, 92 Biological Process GO terms, many of which are related to transcriptional activity, were also estimated to have reduced miRNA repression in both mLi and mLu. Among the KEGG pathways, only mLu had pathways predicted to be less repressed, including Long Term Potentiation, Axon Guidance, Long Term Depression, Ubiquitin Mediated Proteolysis, Neurotrophin Signaling Pathway, TGF Beta Signaling Pathway, Adherens Junction and Phosphatidylinositol Signaling System. One KEGG pathway, Systemic Lupus Erythematosus, was estimated to be more repressed in both mLi and mLu, while the Parkinsons Disease KEGG pathway was repressed only in mLu. Notably, no differentially repressed GO terms or KEGG pathways were shared between the PM datasets and the other two sites. There were also many GO terms and KEGG pathways that were differentially repressed specifically to individual sites. All results for the GSE are shown in Supplementary File S6.

## DISCUSSION

The initial differential expression analysis comparing pCRC and nCR revealed 68 differentially expressed miRNAs, reflecting major and genome-wide changes of malignant transformation. Among the up-regulated miRNAs, a subset representing previously reported ‘oncoMirs’ was identified, including Mir-21, and members of the three clusters Mir-17–92, Mir-31 and Mir-221. These miRNAs have validated mRNA targets in CRC, such as phosphatase and tensin homolog (PTEN), transforming growth factor beta receptor (TGFBR) and Smad (25,26,39). A number of other miRNAs previously connected to CRC were also up-regulated, such as MIR-15 family member Mir-29-P2a/P2b (*miR-29b*), two MIR-96 family members, Mir-135-P3, Mir-130-P2a (*miR-301a*) (40,41), Mir-181-P1c (42) and Mir-224 (25,26). One up-regulated miRNA, Mir-95-P2 (*miR-421*), has not been reported for CRC previously, but its deregulation has been demonstrated in other cancers (43). The remaining down-regulated miRNAs were all previously reported to be associated with CRC; for instance, the tumor suppressor MIR-10 family (4 genes), MIR-15, MIR-192 and MIR-194 (25,26) have been implicated in CRC progression. The identification of miRNAs previously reported to be deregulated in CRC and associated with relevant signaling pathways provides confidence in our analytical approach.

Using the same analytical strategy, alterations in miRNA expression was identified, which included five miRNAs that were differentially expressed in at least two metastatic sites compared to pCRC. Of these, Mir-210_3p and Mir-191_5p were up-regulated at all three metastatic sites, which would suggest a strong metastasis-related significance. Mir-210_3p is in the literature known as the ‘hypoxamiR’ because of its key involvement in the cellular response to hypoxia. The Mir-210_3p promoter has a binding site for hypoxia inducible transcription factors HIF-1$\alpha$ and HIF-2$\alpha \,\,$(44) which coordinate cellular responses to hypoxic stress, including regulation of key pathways in metastasis, such as angiogenesis, cell proliferation, differentiation and apoptosis (45). Increased Mir-210_3p expression, which was additionally validated by qPCR in a separate set of samples, therefore suggests hypoxic stress to be a common feature of CRC metastasis, irrespective of metastatic site. In contrast, the role of Mir-191_5p in metastasis is less well established, and this miRNA is therefore an interesting candidate for further follow-up studies. Additionally, three miRNAs, Mir-8-P1b_3p, Mir-1307_5p and Mir-155_5p, were up-regulated at two of three metastatic sites. Mir-8-P1b_3p (*miR-141*) is a member of the MIR-8 (*miR-200*) family, which is strongly involved in epithelial-to-mesenchymal transition (EMT) and targets transcription factors ZEB1 and ZEB2, which in turn suppress E-cadherin expression (46,47). In our data, Mir-8-P1b_3p was down-regulated in pCRC relative to nCR, while up-regulated in mLi and mLu (Figure 3). This fits well with the concept that tumors undergo EMT as part of tumorigenesis at the primary site, while in the established metastasis the inverse process, mesenchymal-to-epithelial transition, is necessary to establish growth in the new metastatic microenvironment. Mir-155_5p, which was preferentially expressed in mLu and PM, is reported to be specifically expressed in lymphocytes and macrophages (16), possibly indicating higher abundance of these cell types in the metastases compared to pCRC. Less is known about the biological activity of Mir-1307_5p, although reports have suggested a role in lung adenocarcinoma proliferation (48) and as a predictor of hepatocellular carcinoma metastasis (49). The identification of this miRNA as highly expressed in mCRC points to its involvement in metastasis, and Mir-1307_5p therefore represents another target for further studies. Several of the differentially expressed miRNAs have previously been implicated in the metastatic process, providing evidence that their observed up-regulation represents adaptations to survival at the metastatic site.

To further investigate potential biological implications of miRNA expression, GSE analyses were performed to explore predicted effects on mRNA expression based on miRNAs that were differentially expressed at the metastatic sites. Through these analyses, GO terms related to transcription were estimated to be less repressed at the metastatic sites, and KEGG pathways ‘Axon Guidance’, ‘Long-Term Potentiation’ and ‘Long-Term Depression’ were estimated to be less repressed in mLu, possibly representing adaptations to challenges in this microenvironment. However, the biology of mRNA repression by miRNAs is complex. The seed sequence of any miRNA can usually target a large number of mRNAs, and an mRNA can potentially be targeted for repression by multiple miRNAs (11). The functional role of these miRNAs will therefore be dependent on the local cellular contexture, and because the net effect on mRNA expression is highly unpredictable, GSE analysis cannot replace experimental validation. Furthermore, a miRNA may potentially have abnormal expression in multiple diseases, such as Mir-21, which has been shown to be up-regulated in 29 different diseases in addition to CRC (50). Therefore, the functional role of the differentially expressed miRNAs is still not clear, but this work still represents an excellent starting point for further studies, as well as a basis for interpreting the, often contradictory, existing literature.

In order to obtain results that are physiologically relevant, the absolute expression level of a miRNA is an important consideration. A cut-off level of 100 RPM was therefore applied as a minimum expression level to be included in these analyses (14). Several of the differentially expressed miRNAs were expressed at very high absolute levels, for instance with Mir-191_5p, Mir-8-P1b_3p and Mir-10-P1a_5p all being expressed at >1000 RPM in both pCRC and mCRC. Expression levels of this magnitude strongly suggest that alterations in expression of these miRNAs will influence target mRNA levels. Mir-10-P1a_5p is of particular interest, being highly and differentially expressed in mLi compared to pCRC (164 925 RPM and 97 123 RPM, respectively), suggesting specific adaptations to the unique conditions of this microenvironment. Previous experimental evidence suggests that the Mir-10-P1a_5p paralogue, Mir-10-P1b_5p, is associated with enhanced metastatic capability by down-regulation of metastasis suppressor Hoxd10 (51–53). Since Mir-10-P1a_5p and Mir-10-P1b_5p share the same seed sequence (ACCCUGU), the range of mRNA targets and functional roles would be expected to be similar.

In clinical cancer studies, the most commonly available material will be bulk tissue samples, and a tumor biopsy will therefore contain a variable amount of cancer cells of epithelial origin together with a mixture of cell types from the ‘host tissue’ (such as fibroblasts, endothelial cells, myocytes, blood cells and immune cells). The importance of keeping cell specificity in mind when interpreting miRNA expression levels is illustrated by the high levels of hepatocyte specific Mir-122_5p in liver metastases compared to pCRC, which is likely due to the presence of hepatocytes in the metastatic tissues, and do not indicate that this is a metastasis biomarker. Similarly, Mir-143_3p/Mir-145_5p were previously suggested to be tumor suppressors due to apparent ‘down-regulation’ in pCRC relative to nCR (54). In our datasets, both miRNAs were detected at much lower levels in pCRC relative to nCR, but since these miRNAs are exclusively expressed in mesenchymal cells, the low expression in pCRC can only be explained by mesenchymal cells being less frequently present in the colorectal tumors than in the normal colorectal wall (55,56). Expression of known cell-type specific miRNAs could also inform on the relative composition of cell types in the different tissues, and as expected, the three tumor adjacent tissues analyzed clustered separately and had distinct miRNA expression patterns. For instance, the epithelial specific miRNAs, such as Mir-8-P2a_3p (miR-200b) and Mir-8-P2b_3p (miR-200c), had similar levels in the malignant tissues, which is consistent with the intestinal epithelial origin of the cancer cells. This means that considering background expression was a necessary step to ensure that cell composition effects would not confound the differential expression analysis, and failure to do so would likely have led to incorrect identification of miRNAs not associated with metastasis. Taken together, when analyzing bulk tissue samples, it is therefore necessary to consider the composition of the ‘host tissue’, which in this study was handled by defining and correcting for background expression and by analyzing tumor samples from different metastatic sites as separate entities.

In addition to applying a 100 RPM cut-off level, correcting for background expression, and analyzing metastatic locations as separate entities, a number of other methodological improvements were implemented in this work. A systematic effort was made to ensure the quality of the analyses and making the data and the analytical pipeline transparent and available to other researchers in the interest of reproducibility. A novel quality assessment step was introduced in the analysis, using the recently developed miRTrace algorithm, resulting in the exclusion of two previous studies (28,38). Furthermore, our recently established database MirGeneDB, containing only *bona fide* miRNA genes, was used as a reference for mapping miRNA reads to the human genome, representing a further level of precision compared to the previously more commonly used repository, miRBase (57). Regretfully, miRBase has been shown to contain a proportion of incorrect miRNA annotations (13), and including such sequences in the analyses could potentially lead to weakening the power of statistical analyses and result in misleading conclusions. Furthermore, to facilitate reproducibility, a common set of bioinformatics tools were used, and raw NGS datasets from all studies were run through an identical pipeline, from postprocessing and read alignment to miRNA gene counting, using the latest version of miRge (23), a bioinformatics pipeline specifically developed with miRNAs in mind. Combining all these measures and specialized tools, the resulting set of differentially expressed miRNAs between pCRC and mCRC therefore represents an excellent starting point for our evolving understanding of miRNA alterations in metastasis.

In summary, this work represents the most comprehensive attempt to characterize miRNA expression in mCRC. An unbiased, stringent and transparent bioinformatics approach was developed and applied to a large compilation of new and previously published NGS datasets to identify miRNAs associated with metastatic progression in CRC. Comparison of pCRC and nCR replicated many previous findings of up- and down-regulation of well-known oncomiRs and tumor-suppressor miRNAs, supporting the analytical strategy. Correction for background expression was performed, and tumor samples from different metastatic sites were analyzed as separate entities using a 100 RPM expression cut-off level to ensure biological relevance. Five miRNAs that were up-regulated at multiple metastatic sites were identified along with a number of miRNAs differentially expressed at individual metastatic sites. Many of these miRNAs have previously been implicated as key players in the metastatic process, while for others, the involvement in tumor cell adaptations at the distant site represent novel findings. The use of open science practices and the biological relevance of the findings lend confidence in the resulting miRNA alterations in mCRC, which provides a starting point for further elucidation of the role of miRNAs in metastatic progression. The main limitations in this work are related to the complexity of miRNA biology as previously discussed, and to the still modest number of available miRNA datasets from mCRC. These factors still limit the possibility to fully interpret the findings in a clinical setting. The pipeline developed for this analysis is freely available to other researchers to expand on the results presented in this work, as well for exploring other cancer entities and disease settings. This work could therefore represent a first step to generate a miRNA tissue expression ‘atlas’ for researchers interested in miRNA biomarkers, for instance to identify miRNAs that are specifically deregulated in a disease of interest.

## DATA AVAILABILITY

Data from current and previous studies can be found at EGA accession number EGAS00001001127 (19), GEO accession numbers: GSE57381 (30), GSE46622 (27) and GSE63119 (28), and SRA accession PRJNA397121 for datasets prepared in this study and (29). The bioinformatics pipeline, singularity containers with the necessary tools, along with a tutorial with example datasets, can be found at (https://github.com/eirikhoye/mirna_pipeline).

## SUPPLEMENTARY DATA

Supplementary Data are available at NAR Cancer Online.

## Supplementary Material

zcab051_Supplemental_Files
