# Supplementary material for: A comprehensive framework for analysis of microRNA sequencing data in metastatic colorectal cancer
Source: NAR Cancer. 2022 Jan 14;4(1):zcab051. doi: 10.1093/narcan/zcab051 (PMC8759566; doi:10.1093/narcan/zcab051)
Supplement: zcab051_Supplemental_Files [file zcab051_supplemental_files.zip › Supplementary table_1.docx]

Supplementary Table 1

| Variable | | |  | | pCRC | | mLi | mLu | PM | | |
| --- | --- | --- | --- | --- | --- | --- | --- | --- | --- | --- | --- |
| Gender; number (%) | | | Male | | 53 (43) | | 25 (66) | 19 (70) | 18 (67) | | |
|  | | | Female | | 69 (57) | | 13 (34) | 8 (30) | 9 (33) | | |
| Age; median (IQR) | | |  | | 70 (61-77) | | 66 (58-72) | 69 (58-74) | 63 (54-70) | | |
| Overall survival; months (median (IQR)) | | |  | | 102 (48-111) * | | 55 (42-64) ** | 80 (30-97) ** | 32 (18-54) *** | | |
| TNM; number (%) | | |  | |  | |  |  |  | | |
|  | | | T1-T2 | | 15 (12) | |  |  |  | | |
|  | | | T3 | | 92 (75) | |  |  |  | | |
|  | | | T4 | | 15 (12) | |  |  |  | | |
|  | | | N0 | | 60 (70) | |  |  |  | | |
|  | | | N1 | | 15 (17) | |  |  |  | | |
|  | | | N2 | | 11 (13) | |  |  |  | | |
|  | | | M1 | | 37 (30) | |  |  |  | | |
| Tumor content; % | | |  | |  | |  |  |  | | |
| This Study | | | Number of cases | |  | | 19 | 22 | 16 |  |  |
|  | | | Median (IQR) | |  | | 60 (50-73) | 50 (50-50) | 50 (48-50) | |  |
| Schee et al | | | Number of cases | | 85 | |  |  |  | |  |
|  | | | Median (IQR) | | 50 (40-60) | |  |  |  | |  |
| Neerincx et al | | | Number of cases >70% | | 31 | | 17 | 2 | 10 | |  |
|  | | | Number of cases <70% | | 5 | | 1 | 2 | 2 | |  |
|  |  |  | |  | |  |  |  |  |  |  |

* Overall and metastasis-free survival was calculated from date of surgery until date of death or diagnosis of metastasis

** Overall survival was calculated from date of metastasis surgery until date of death

*** Overall survival was calculated from date of HIPEC or surgery until date of death

Inter Quartile Range (IQR); Tumor Node Metastasis (TNM); primary colorectal cancer (pCRC); CRC liver metastasis (mLi; CRC lung metastasis (mLu); CRC peritoneal metastasis (PM)
