# Supplementary material for: A comprehensive framework for analysis of microRNA sequencing data in metastatic colorectal cancer
Source: NAR Cancer. 2022 Jan 14;4(1):zcab051. doi: 10.1093/narcan/zcab051 (PMC8759566; doi:10.1093/narcan/zcab051)
Supplement: zcab051_Supplemental_Files [file zcab051_supplemental_files.zip › Supplementary_Material_Captions.docx]

**Supplementary material**

Supplementary file 1: miRTrace QC reports (.html)

Supplementary file 2: miRge3.0 count matrix and sample metadata (.csv)

Supplementary file 3: R-markdown: DESeq2 differential expression analysis (.html)

Supplementary file 4: R-markdown: code for figures (.html)

Supplementary file 5: R-markdown qPCR analysis (.html)

Supplementary file 6: R-markdown GSE results (.html)

Supplementary file 7: Raw qPCR data (.csv and .py)

Supplementary file 8: Supplementary Table 1 (.wordx)
