# Supplementary material for: A comprehensive framework for analysis of microRNA sequencing data in metastatic colorectal cancer
Source: NAR Cancer. 2022 Jan 14;4(1):zcab051. doi: 10.1093/narcan/zcab051 (PMC8759566; doi:10.1093/narcan/zcab051)
Supplement: zcab051_Supplemental_Files [file zcab051_supplemental_files.zip › Supplementary_Files.zip!/Supplementary_file_6_GSE_results.zip!/Supplementary_file_6_GSE_results/supplementary_file_6_GSE_results.html]

RBiomirGS


# RBiomirGS

#### Eirik Høye

#### 2021-02-08

Input is Deseq2 mCRC versus pCRC Log2Fold Change (LFC) and p-values adjusted for multiple hypothesis testing (padj).

Differential expression is on individual metastatic sites

To account for the background noise of normal adjacent cells present in the metastatic tissue samples, if the normal background LFC is in the same direction, the mCRC.vs.pCRC LFC is subtracted the normal background LFC, to max/min of 0 LFC. In addition, if the LFCs are in the same direction, the padj value of mCRC.vs.pCRC is added 1 - normal background padj

The Gene Set analysis is done using the RBiomirGS R package: https://github.com/jzhangc/git\_RBiomirGS

```
library(RBiomirGS)
library(tidyverse)
```

```
mirgenedb2mirbase <- read_delim('/Users/eirikhoy/Dropbox/projects/comet_analysis/data/hsa_MirGeneDB_to_miRBase.csv', delim=';')
mirgenedb2mirbase <- mirgenedb2mirbase %>% select(MirGeneDB_ID, MiRBase_ID)
mirgenedb2mirbase$MirGeneDB_ID <- str_remove(mirgenedb2mirbase$MirGeneDB_ID, '-v[0-9]')
mirgenedb2mirbase <- mirgenedb2mirbase %>% distinct(MirGeneDB_ID, MiRBase_ID)
```

```
raw_mli <- read_csv('/Users/eirikhoy/Dropbox/projects/comet_analysis/data/Deseq_result_clm_vs_pcrc.csv')

remove_5p3p <- function(x){
  return(str_split_fixed(x, pattern='_', n=2)[,1])
}
tmp <- as.data.frame(apply(raw_mli, MARGIN=2, FUN=remove_5p3p))

raw_mli$MirGeneDB_ID <- tmp$miRNA

new_raw_mli <- left_join(raw_mli, mirgenedb2mirbase, by='MirGeneDB_ID')
raw_mli <- new_raw_mli %>% drop_na() %>% select(MiRBase_ID, LFC_adj_background, padj)
raw_mli
```

```
## # A tibble: 387 x 3
##    MiRBase_ID   LFC_adj_background     padj
##    <chr>                     <dbl>    <dbl>
##  1 hsa-let-7a-2             0      8.87e- 1
##  2 hsa-let-7e               0      8.99e- 1
##  3 hsa-let-7c               0      4.78e- 1
##  4 hsa-let-7f-2            -0.0668 5.90e- 1
##  5 hsa-let-7f-1            -0.0221 9.07e- 1
##  6 hsa-let-7b              -0.131  3.61e- 1
##  7 hsa-mir-98               0      8.55e- 2
##  8 hsa-let-7d               0.0153 9.61e- 1
##  9 hsa-let-7i              -0.373  1.69e-14
## 10 hsa-let-7g              -0.151  8.24e- 2
## # … with 377 more rows
```

```
raw_mlu <- read_csv('/Users/eirikhoy/Dropbox/projects/comet_analysis/data/Deseq_result_mlu_vs_pcrc.csv')

remove_5p3p <- function(x){
  return(str_split_fixed(x, pattern='_', n=2)[,1])
}
tmp <- as.data.frame(apply(raw_mlu, MARGIN=2, FUN=remove_5p3p))

raw_mlu$MirGeneDB_ID <- tmp$miRNA

new_raw_mlu <- left_join(raw_mlu, mirgenedb2mirbase, by='MirGeneDB_ID')
raw_mlu <- new_raw_mlu %>% drop_na() %>% select(MiRBase_ID, LFC_adj_background, padj)
raw_mlu
```

```
## # A tibble: 387 x 3
##    MiRBase_ID   LFC_adj_background         padj
##    <chr>                     <dbl>        <dbl>
##  1 hsa-let-7a-2             -0.371 0.000407    
##  2 hsa-let-7e               -0.865 0.000000255 
##  3 hsa-let-7c                0     0.533       
##  4 hsa-let-7f-2             -0.362 0.0000766   
##  5 hsa-let-7f-1              0     0.757       
##  6 hsa-let-7b               -0.387 0.00122     
##  7 hsa-mir-98               -0.151 0.181       
##  8 hsa-let-7d               -0.272 0.0226      
##  9 hsa-let-7i               -0.308 0.0206      
## 10 hsa-let-7g                0     0.0000000593
## # … with 377 more rows
```

```
raw_pm <- read_csv('/Users/eirikhoy/Dropbox/projects/comet_analysis/data/Deseq_result_pc_vs_pcrc.csv')

remove_5p3p <- function(x){
  return(str_split_fixed(x, pattern='_', n=2)[,1])
}
tmp <- as.data.frame(apply(raw_pm, MARGIN=2, FUN=remove_5p3p))

raw_pm$MirGeneDB_ID <- tmp$miRNA

new_raw_pm <- left_join(raw_pm, mirgenedb2mirbase, by='MirGeneDB_ID')
raw_pm <- new_raw_pm %>% drop_na() %>% select(MiRBase_ID, LFC_adj_background, padj)
raw_pm
```

```
## # A tibble: 372 x 3
##    MiRBase_ID   LFC_adj_background       padj
##    <chr>                     <dbl>      <dbl>
##  1 hsa-let-7a-2            -0.195  0.0901    
##  2 hsa-let-7e              -0.109  0.370     
##  3 hsa-let-7c               0      0.00000140
##  4 hsa-let-7f-2            -0.236  0.0142    
##  5 hsa-let-7f-1            -0.0276 0.848     
##  6 hsa-let-7b              -0.153  0.263     
##  7 hsa-mir-98              -0.189  0.000416  
##  8 hsa-let-7d              -0.120  0.00621   
##  9 hsa-let-7i              -0.142  0.324     
## 10 hsa-let-7g              -0.0427 0.000416  
## # … with 362 more rows
```

```
                                                # Get all targets
```

```
# Only needs to be run once
rbiomirgs_mrnascan(objTitle = "miRNA", 
                   mir = raw_mli$MiRBase_ID, 
                   sp = 'hsa', 
                   queryType = 'predicted', 
                   parallelComputing = TRUE, 
                   clusterType = "PSOCK")
```

```
                                                # Cellular Component
```

```
# mLi
raw_m <- as.data.frame(raw_mli)
rbiomirgs_logistic(objTitle = 'cc_mli_mirna_mrna_iwls',
                   mirna_DE = raw_m, 
                   var_mirnaName = 'MiRBase_ID',
                   var_mirnaFC = 'LFC_adj_background', 
                   var_mirnaP = 'padj', 
                   mrnalist = miRNA_mrna_entrez_list, 
                   mrna_Weight = NULL, 
                   #gs_file = '/Users/eirikhoy/Dropbox/projects/comet_analysis/data/gmt_files/Human_GOBP_AllPathways_no_GO_iea_January_13_2021_symbol.gmt',
                   gs_file = '/Users/eirikhoy/Dropbox/projects/comet_analysis/data/gmt_files/c5.go.cc.v7.2.entrez.gmt',
                   optim_method = "IWLS", 
                   p.adj = "fdr",
                   parallelComputing = TRUE, 
                   clusterType = "PSOCK")

rbiomirgs_bar(gsadfm = cc_mli_mirna_mrna_iwls_GS, gs.name = TRUE, n = 15, 
                    y.rightside = FALSE, yTxtSize = 8, plotWidth = 200, 
                    plotHeight = 200, xLabel = "Log Odds Ratio", 
                    signif_only = TRUE)
```

```
rbiomirgs_volcano(gsadfm = cc_mli_mirna_mrna_iwls_GS, topgsLabel = TRUE, n = 15, gsLabelSize = 3,
                  sigColour = 'blue', plotWidth = 200, plotHeight = 180,
                  xLabel = "Log Odds Ratio")
```

```
write_csv(x = mirnascore, file = "cc_mli_mirnascore")
write_csv(x = mrnascore, file = "cc_mli_mrnascore")
```

```
# mLu
raw_m <- as.data.frame(raw_mlu)
rbiomirgs_logistic(objTitle = 'cc_mlu_mirna_mrna_iwls',
                   mirna_DE = raw_m, 
                   var_mirnaName = 'MiRBase_ID',
                   var_mirnaFC = 'LFC_adj_background', 
                   var_mirnaP = 'padj', 
                   mrnalist = miRNA_mrna_entrez_list, 
                   mrna_Weight = NULL, 
                   #gs_file = '/Users/eirikhoy/Dropbox/projects/comet_analysis/data/gmt_files/Human_GOBP_AllPathways_no_GO_iea_January_13_2021_symbol.gmt',
                   gs_file = '/Users/eirikhoy/Dropbox/projects/comet_analysis/data/gmt_files/c5.go.cc.v7.2.entrez.gmt',
                   optim_method = "IWLS", 
                   p.adj = "fdr",
                   parallelComputing = TRUE, 
                   clusterType = "PSOCK")

rbiomirgs_bar(gsadfm = cc_mlu_mirna_mrna_iwls_GS, gs.name = TRUE, n = 15, 
                    y.rightside = FALSE, yTxtSize = 8, plotWidth = 200, 
                    plotHeight = 200, xLabel = "Log Odds Ratio", 
                    signif_only = TRUE)
```

```
rbiomirgs_volcano(gsadfm = cc_mlu_mirna_mrna_iwls_GS, topgsLabel = TRUE, n = 15, gsLabelSize = 3,
                  sigColour = 'blue', plotWidth = 200, plotHeight = 180,
                  xLabel = "Log Odds Ratio")
```

```
write_csv(x = mirnascore, file = "cc_mlu_mirnascore")
write_csv(x = mrnascore, file = "cc_mlu_mrnascore")
```

```
# PM
raw_m <- as.data.frame(raw_pm)
rbiomirgs_logistic(objTitle = 'cc_pm_mirna_mrna_iwls',
                   mirna_DE = raw_m, 
                   var_mirnaName = 'MiRBase_ID',
                   var_mirnaFC = 'LFC_adj_background', 
                   var_mirnaP = 'padj', 
                   mrnalist = miRNA_mrna_entrez_list, 
                   mrna_Weight = NULL, 
                   #gs_file = '/Users/eirikhoy/Dropbox/projects/comet_analysis/data/gmt_files/Human_GOBP_AllPathways_no_GO_iea_January_13_2021_symbol.gmt',
                   gs_file = '/Users/eirikhoy/Dropbox/projects/comet_analysis/data/gmt_files/c5.go.cc.v7.2.entrez.gmt',
                   optim_method = "IWLS", 
                   p.adj = "fdr",
                   parallelComputing = TRUE, 
                   clusterType = "PSOCK")

rbiomirgs_bar(gsadfm = cc_pm_mirna_mrna_iwls_GS, gs.name = TRUE, n = 15, 
                    y.rightside = FALSE, yTxtSize = 8, plotWidth = 200, 
                    plotHeight = 200, xLabel = "Log Odds Ratio", 
                    signif_only = TRUE)
```

```
rbiomirgs_volcano(gsadfm = cc_pm_mirna_mrna_iwls_GS, topgsLabel = TRUE, n = 15, gsLabelSize = 3,
                  sigColour = 'blue', plotWidth = 200, plotHeight = 180,
                  xLabel = "Log Odds Ratio")
```

```
write_csv(x = mirnascore, file = "cc_pm_mirnascore")
write_csv(x = mrnascore, file = "cc_pm_mrnascore")
```

```
                                                # Biological Process
```

```
raw_m <- as.data.frame(raw_mli)
rbiomirgs_logistic(objTitle = 'bp_mli_mirna_mrna_iwls',
                   mirna_DE = raw_m, 
                   var_mirnaName = 'MiRBase_ID',
                   var_mirnaFC = 'LFC_adj_background', 
                   var_mirnaP = 'padj', 
                   mrnalist = miRNA_mrna_entrez_list, 
                   mrna_Weight = NULL, 
                   #gs_file = '/Users/eirikhoy/Dropbox/projects/comet_analysis/data/gmt_files/Human_GOBP_AllPathways_no_GO_iea_January_13_2021_symbol.gmt',
                   gs_file = '/Users/eirikhoy/Dropbox/projects/comet_analysis/data/gmt_files/c5.go.bp.v7.2.entrez.gmt',
                   optim_method = "IWLS", 
                   p.adj = "fdr",
                   parallelComputing = TRUE, 
                   clusterType = "PSOCK")

rbiomirgs_bar(gsadfm = bp_mli_mirna_mrna_iwls_GS, gs.name = TRUE, n = 15, 
                    y.rightside = FALSE, yTxtSize = 8, plotWidth = 250, 
                    plotHeight = 300, xLabel = "Log Odds Ratio", 
                    signif_only = TRUE)
```

```
rbiomirgs_volcano(gsadfm = bp_mli_mirna_mrna_iwls_GS, topgsLabel = TRUE, n = 15, gsLabelSize = 3,
                  sigColour = 'blue', plotWidth = 250, plotHeight = 200,
                  xLabel = 'Log Odds Ratio')
```

```
write_csv(x = mirnascore, file = "bp_mli_mirnascore")
write_csv(x = mrnascore, file = "bp_mli_mrnascore")
```

```
raw_m <- as.data.frame(raw_mlu)
rbiomirgs_logistic(objTitle = 'bp_mlu_mirna_mrna_iwls',
                   mirna_DE = raw_m, 
                   var_mirnaName = 'MiRBase_ID',
                   var_mirnaFC = 'LFC_adj_background', 
                   var_mirnaP = 'padj', 
                   mrnalist = miRNA_mrna_entrez_list, 
                   mrna_Weight = NULL, 
                   #gs_file = '/Users/eirikhoy/Dropbox/projects/comet_analysis/data/gmt_files/Human_GOBP_AllPathways_no_GO_iea_January_13_2021_symbol.gmt',
                   gs_file = '/Users/eirikhoy/Dropbox/projects/comet_analysis/data/gmt_files/c5.go.bp.v7.2.entrez.gmt',
                   optim_method = "IWLS", 
                   p.adj = "fdr",
                   parallelComputing = TRUE, 
                   clusterType = "PSOCK")

rbiomirgs_bar(gsadfm = bp_mlu_mirna_mrna_iwls_GS, gs.name = TRUE, n = 15, 
                    y.rightside = FALSE, yTxtSize = 8, plotWidth = 400, 
                    plotHeight = 300, xLabel = "Log Odds Ratio", 
                    signif_only = TRUE)
```

```
rbiomirgs_volcano(gsadfm = bp_mlu_mirna_mrna_iwls_GS, topgsLabel = TRUE, n = 15, gsLabelSize = 3,
                  sigColour = 'blue', plotWidth = 450, plotHeight = 200,
                  xLabel = 'Log Odds Ratio')
```

```
write_csv(x = mirnascore, file = "bp_mlu_mirnascore")
write_csv(x = mrnascore, file  = "bp_mlu_mrnascore")
```

```
raw_m <- as.data.frame(raw_pm)
rbiomirgs_logistic(objTitle = 'bp_pm_mirna_mrna_iwls',
                   mirna_DE = raw_m, 
                   var_mirnaName = 'MiRBase_ID',
                   var_mirnaFC = 'LFC_adj_background', 
                   var_mirnaP = 'padj', 
                   mrnalist = miRNA_mrna_entrez_list, 
                   mrna_Weight = NULL, 
                   #gs_file = '/Users/eirikhoy/Dropbox/projects/comet_analysis/data/gmt_files/Human_GOBP_AllPathways_no_GO_iea_January_13_2021_symbol.gmt',
                   gs_file = '/Users/eirikhoy/Dropbox/projects/comet_analysis/data/gmt_files/c5.go.bp.v7.2.entrez.gmt',
                   optim_method = "IWLS", 
                   p.adj = "fdr",
                   parallelComputing = TRUE, 
                   clusterType = "PSOCK")

rbiomirgs_bar(gsadfm = bp_pm_mirna_mrna_iwls_GS, gs.name = TRUE, n = 15, 
                    y.rightside = FALSE, yTxtSize = 8, plotWidth = 450, 
                    plotHeight = 300, xLabel = "Log Odds Ratio", 
                    signif_only = TRUE)
```

```
rbiomirgs_volcano(gsadfm = bp_pm_mirna_mrna_iwls_GS, topgsLabel = TRUE, n = 15, gsLabelSize = 3,
                  sigColour = 'blue', plotWidth = 450, plotHeight = 200,
                  xLabel = 'Log Odds Ratio')
```

```
write_csv(x = mirnascore, file = "bp_pm_mirnascore")
write_csv(x = mrnascore, file = "bp_pm_mrnascore")
```

```
                                                # Molecular Function
```

```
raw_m <- as.data.frame(raw_mli)
rbiomirgs_logistic(objTitle = 'mf_mli_mirna_mrna_iwls',
                   mirna_DE = raw_m, 
                   var_mirnaName = 'MiRBase_ID',
                   var_mirnaFC = 'LFC_adj_background', 
                   var_mirnaP = 'padj', 
                   mrnalist = miRNA_mrna_entrez_list, 
                   mrna_Weight = NULL, 
                   #gs_file = '/Users/eirikhoy/Dropbox/projects/comet_analysis/data/gmt_files/Human_GOBP_AllPathways_no_GO_iea_January_13_2021_symbol.gmt',
                   gs_file = '/Users/eirikhoy/Dropbox/projects/comet_analysis/data/gmt_files/c5.go.mf.v7.2.entrez.gmt',
                   optim_method = "IWLS", 
                   p.adj = "fdr",
                   parallelComputing = TRUE, 
                   clusterType = "PSOCK")

rbiomirgs_bar(gsadfm = mf_mli_mirna_mrna_iwls_GS, gs.name = TRUE, n = 15, 
                    y.rightside = FALSE, yTxtSize = 8, plotWidth = 200, 
                    plotHeight = 150, xLabel = "Log odss ratio", 
                    signif_only = TRUE)
```

```
rbiomirgs_volcano(gsadfm = mf_mli_mirna_mrna_iwls_GS, topgsLabel = TRUE, n = 15, gsLabelSize = 3,
                  sigColour = 'blue', plotWidth = 250, plotHeight = 200,
                  xLabel = 'Log Odds Ratio')
```

```
write_csv(x = mirnascore, file = "mf_mli_mirnascore")
write_csv(x = mrnascore, file  = "mf_mli_mrnascore")
```

```
raw_m <- as.data.frame(raw_mlu)
rbiomirgs_logistic(objTitle = 'mf_mlu_mirna_mrna_iwls',
                   mirna_DE = raw_m, 
                   var_mirnaName = 'MiRBase_ID',
                   var_mirnaFC = 'LFC_adj_background', 
                   var_mirnaP = 'padj', 
                   mrnalist = miRNA_mrna_entrez_list, 
                   mrna_Weight = NULL, 
                   #gs_file = '/Users/eirikhoy/Dropbox/projects/comet_analysis/data/gmt_files/Human_GOBP_AllPathways_no_GO_iea_January_13_2021_symbol.gmt',
                   gs_file = '/Users/eirikhoy/Dropbox/projects/comet_analysis/data/gmt_files/c5.go.mf.v7.2.entrez.gmt',
                   optim_method = "IWLS", 
                   p.adj = "fdr",
                   parallelComputing = TRUE, 
                   clusterType = "PSOCK")

rbiomirgs_bar(gsadfm = mf_mlu_mirna_mrna_iwls_GS, gs.name = TRUE, n = 15, 
                    y.rightside = FALSE, yTxtSize = 8, plotWidth = 200, 
                    plotHeight = 150, xLabel = "Log odss ratio", 
                    signif_only = TRUE)
```

```
rbiomirgs_volcano(gsadfm = mf_mlu_mirna_mrna_iwls_GS, topgsLabel = TRUE, n = 15, gsLabelSize = 3,
                  sigColour = 'blue', plotWidth = 250, plotHeight = 200,
                  xLabel = 'Log Odds Ratio')
```

```
write_csv(x = mirnascore, file = "mf_mlu_mirnascore")
write_csv(x = mrnascore, file  = "mf_mlu_mrnascore")
```

```
raw_m <- as.data.frame(raw_pm)
rbiomirgs_logistic(objTitle = 'mf_pm_mirna_mrna_iwls',
                   mirna_DE = raw_m, 
                   var_mirnaName = 'MiRBase_ID',
                   var_mirnaFC = 'LFC_adj_background', 
                   var_mirnaP = 'padj', 
                   mrnalist = miRNA_mrna_entrez_list, 
                   mrna_Weight = NULL, 
                   #gs_file = '/Users/eirikhoy/Dropbox/projects/comet_analysis/data/gmt_files/Human_GOBP_AllPathways_no_GO_iea_January_13_2021_symbol.gmt',
                   gs_file = '/Users/eirikhoy/Dropbox/projects/comet_analysis/data/gmt_files/c5.go.mf.v7.2.entrez.gmt',
                   optim_method = "IWLS", 
                   p.adj = "fdr",
                   parallelComputing = TRUE, 
                   clusterType = "PSOCK")

rbiomirgs_bar(gsadfm = mf_pm_mirna_mrna_iwls_GS, gs.name = TRUE, n = 15, 
                    y.rightside = FALSE, yTxtSize = 8, plotWidth = 200, 
                    plotHeight = 150, xLabel = "Log odss ratio", 
                    signif_only = TRUE)
```

```
rbiomirgs_volcano(gsadfm = mf_pm_mirna_mrna_iwls_GS, topgsLabel = TRUE, n = 15, gsLabelSize = 3,
                  sigColour = 'blue', plotWidth = 250, plotHeight = 200,
                  xLabel = 'Log Odds Ratio')
```

```
write_csv(x = mirnascore, file = "mf_pm_mirnascore")
write_csv(x = mrnascore, file  = "mf_pm_mrnascore")
```

```
                                                # KEGG
```

```
raw_m <- as.data.frame(raw_mli)
rbiomirgs_logistic(objTitle = 'kegg_mli_mirna_mrna_iwls',
                   mirna_DE = raw_m, 
                   var_mirnaName = 'MiRBase_ID',
                   var_mirnaFC = 'LFC_adj_background', 
                   var_mirnaP = 'padj', 
                   mrnalist = miRNA_mrna_entrez_list, 
                   mrna_Weight = NULL, 
                   #gs_file = '/Users/eirikhoy/Dropbox/projects/comet_analysis/data/gmt_files/Human_GOBP_AllPathways_no_GO_iea_January_13_2021_symbol.gmt',
                   gs_file = '/Users/eirikhoy/Dropbox/projects/comet_analysis/data/gmt_files/c2.cp.kegg.v7.2.entrez.gmt',
                   optim_method = "IWLS", 
                   p.adj = "fdr",
                   parallelComputing = TRUE, 
                   clusterType = "PSOCK")

rbiomirgs_bar(gsadfm = kegg_mli_mirna_mrna_iwls_GS, gs.name = TRUE, n = 15, 
                    y.rightside = FALSE, yTxtSize = 8, plotWidth = 200, 
                    plotHeight = 50, xLabel = "Log odss ratio", 
                    signif_only = TRUE)
```

```
rbiomirgs_volcano(gsadfm = kegg_mli_mirna_mrna_iwls_GS, topgsLabel = TRUE, n = 15, gsLabelSize = 3,
                  sigColour = 'blue', plotWidth = 160, plotHeight = 120,
                  xLabel = 'model coefficient')
```

```
write_csv(x = mirnascore, file = "kegg_mli_mirnascore")
write_csv(x = mrnascore, file  = "kegg_mli_mrnascore")
```

```
raw_m <- as.data.frame(raw_mlu)
rbiomirgs_logistic(objTitle = 'kegg_mlu_mirna_mrna_iwls',
                   mirna_DE = raw_m, 
                   var_mirnaName = 'MiRBase_ID',
                   var_mirnaFC = 'LFC_adj_background', 
                   var_mirnaP = 'padj', 
                   mrnalist = miRNA_mrna_entrez_list, 
                   mrna_Weight = NULL, 
                   #gs_file = '/Users/eirikhoy/Dropbox/projects/comet_analysis/data/gmt_files/Human_GOBP_AllPathways_no_GO_iea_January_13_2021_symbol.gmt',
                   gs_file = '/Users/eirikhoy/Dropbox/projects/comet_analysis/data/gmt_files/c2.cp.kegg.v7.2.entrez.gmt',
                   optim_method = "IWLS", 
                   p.adj = "fdr",
                   parallelComputing = TRUE, 
                   clusterType = "PSOCK")

rbiomirgs_bar(gsadfm = kegg_mlu_mirna_mrna_iwls_GS, gs.name = TRUE, n = 15, 
                    y.rightside = FALSE, yTxtSize = 8, plotWidth = 200, 
                    plotHeight = 50, xLabel = "Log odss ratio", 
                    signif_only = TRUE)
```

```
rbiomirgs_volcano(gsadfm = kegg_mlu_mirna_mrna_iwls_GS, topgsLabel = TRUE, n = 15, gsLabelSize = 3,
                  sigColour = 'blue', plotWidth = 160, plotHeight = 120,
                  xLabel = 'model coefficient')
```

```
write_csv(x = mirnascore, file = "kegg_mlu_mirnascore")
write_csv(x = mrnascore, file  = "kegg_mlu_mrnascore")
```

```
raw_m <- as.data.frame(raw_pm)
rbiomirgs_logistic(objTitle = 'kegg_pm_mirna_mrna_iwls',
                   mirna_DE = raw_m, 
                   var_mirnaName = 'MiRBase_ID',
                   var_mirnaFC = 'LFC_adj_background', 
                   var_mirnaP = 'padj', 
                   mrnalist = miRNA_mrna_entrez_list, 
                   mrna_Weight = NULL, 
                   #gs_file = '/Users/eirikhoy/Dropbox/projects/comet_analysis/data/gmt_files/Human_GOBP_AllPathways_no_GO_iea_January_13_2021_symbol.gmt',
                   gs_file = '/Users/eirikhoy/Dropbox/projects/comet_analysis/data/gmt_files/c2.cp.kegg.v7.2.entrez.gmt',
                   optim_method = "IWLS", 
                   p.adj = "fdr",
                   parallelComputing = TRUE, 
                   clusterType = "PSOCK")

#rbiomirgs_bar(gsadfm = kegg_pm_mirna_mrna_iwls_GS, gs.name = TRUE, n = 15, 
#                    y.rightside = FALSE, yTxtSize = 8, plotWidth = 200, 
#                    plotHeight = 50, xLabel = "Log odss ratio", 
#                    signif_only = TRUE)

#rbiomirgs_volcano(gsadfm = kegg_pm_mirna_mrna_iwls_GS, topgsLabel = TRUE, n = 15, gsLabelSize = 3,
#                  sigColour = 'blue', plotWidth = 160, plotHeight = 120,
#                  xLabel = 'model coefficient')

write_csv(x = mirnascore, file = "kegg_pm_mirnascore")
write_csv(x = mrnascore, file  = "kegg_pm_mrnascore")
```
